# Conformational dynamics is key to understanding loss-of-function of NQO1 cancer-associated polymorphisms and its correction by pharmacological ligands

**DOI:** 10.1038/srep20331

**Published:** 2016-02-03

**Authors:** Encarnación Medina-Carmona, Rogelio J. Palomino-Morales, Julian E. Fuchs, Esperanza Padín-Gonzalez, Noel Mesa-Torres, Eduardo Salido, David J. Timson, Angel L. Pey

**Affiliations:** 1 Department of Physical Chemistry, Faculty of Sciences, University of Granada, Granada, Spain; 2 Department of Biochemistry and Molecular Biology I, Faculty of Sciences, University of Granada, Granada, Spain; 3 Centre for Molecular Informatics, Department of Chemistry, University of Cambridge, Cambridge, UK; 4 Hospital Universitario de Canarias, Tenerife, Spain; 5 School of Biological Sciences, Queen´s University Belfast, Belfast, UK; 6 School of Pharmacy and Biomolecular Sciences, The University of Brighton, Brighton, UK

## Abstract

Protein dynamics is essential to understand protein function and stability, even though is rarely investigated as the origin of loss-of-function due to genetic variations. Here, we use biochemical, biophysical, cell and computational biology tools to study two loss-of-function and cancer-associated polymorphisms (p.R139W and p.P187S) in human NAD(P)H quinone oxidoreductase 1 (NQO1), a FAD-dependent enzyme which activates cancer pro-drugs and stabilizes several oncosuppressors. We show that p.P187S strongly destabilizes the NQO1 dimer *in vitro* and increases the flexibility of the C-terminal domain, while a combination of FAD and the inhibitor dicoumarol overcome these alterations. Additionally, changes in global stability due to polymorphisms and ligand binding are linked to the dynamics of the dimer interface, whereas the low activity and affinity for FAD in p.P187S is caused by increased fluctuations at the FAD binding site. Importantly, NQO1 steady-state protein levels in cell cultures correlate primarily with the dynamics of the C-terminal domain, supporting a directional preference in NQO1 proteasomal degradation and the use of ligands binding to this domain to stabilize p.P187S *in vivo*. In conclusion, protein dynamics are fundamental to understanding loss-of-function in p.P187S, and to develop new pharmacological therapies to rescue this function.

The study of NAD(P)H quinone oxidoreductase 1 (NQO1, EC 1.6.5.2) polymorphisms is particularly interesting due to its enhanced expression in several types of cancer^1^,^2^,^3^. NQO1 is a dimeric, two-domain FAD-dependent enzyme (Fig. 1A) which catalyses the two electron reduction of quinones and related substrates through an enzyme-substituted mechanism in which NAD(P)H enters the active site, reduces the FAD and exits as the oxidised form, allowing the subsequent substrate binding and reduction by the FADH_2_^4^. Primarily, NQO1 avoids the formation of reactive semiquinones, maintains antioxidants such as α-tocopherol and ubiquinone in their reduced state, and also activates some anticancer bioreductive drugs (e.g. mitomycin C (MMC) and a MMC analogue, EO9)^4^,^5^. Additionally, NQO1 interacts with tumour suppressors such as p53 and p73 and stabilizes them towards proteasomal degradation^6^,^7^,^8^,^9^, while its interaction with the 20S proteasome prevents the degradation of a plethora of proteins with intrinsically disordered regions, including several cell cycle regulators, tumor suppressors and apoptotic proteins^10^.

Two single nucleotide polymorphisms in NQO1 were originally isolated from cancer cell lines, namely rs1800566/c.C609T/p.P187S and rs1131341/c.C465T/p.R139W^11^,^12^,^13^. The frequency of the p.P187S allele is much higher than of p.R139W in human population (about 30% vs. 1–2%; www.ensemble.org;)^14^. A recent comprehensive meta-analysis using over 21000 cases and 25000 controls have supported an association of the p.P187S polymorphism in homozygosis and an overall increased cancer risk^15^. Although p.R139W is rarely found in homozygosis, this polymorphism in heterozygosis has been associated with increased risk of developing acute lymphoblastic leukemia in children^16^. To our knowledge, no exhaustive association studies of these polymorphisms and the response to different chemotherapies have been described so far.

p.P187S is known to strongly reduce cellular NQO1 protein levels and activity^13^,^17^, thus diminishing its efficiency in the reductive activation of cancer drugs such as MMC. The intracellular instability of p.P187S is caused by enhanced fast degradation by the 20S/26S proteasome^18^,^19^ while its low activity is in part associated to its low affinity for FAD^18^,^20^,^21^. The p.R139W polymorphism is less well studied, showing WT activity levels towards using dichlorophenol indophenol (DCPIP) as substrate^20^ but reduced activity towards MMC^11^. This polymorphism is thought to primarily reduce the sensitivity towards MMC in cell lines due to alternative splicing causing skipping of exon 4, and therefore, decreasing the expression of the active full-length enzyme^11^,^22^. Even though p.P187S does not affect the overall conformation of NQO1^20^, a higher population of apo-NQO1 due to its defect in FAD binding may shift the folding equilibrium towards partially denatured states, which are much more sensitive to proteasomal degradation^18^,^20^,^21^. Therefore, boosting intracellular levels of FAD may protect p.P187S towards degradation^18^,^20^,^21^. Remarkably, X-ray crystallographic analysis of p.P187S has revealed no significant structural changes, while nuclear magnetic resonance (NMR) spectroscopy and proteolysis experiments supported partial unfolding and enhanced flexibility of p.P187S in solution^21^.

Protein dynamics is a key factor in many aspects of protein function, such as enzyme catalysis^23^, allosteric regulation^24^ and protein degradation^25^. In the case of NQO1, polymorphisms may affect local dynamics therefore leading to changes in protein stability and functionality, and also, natural ligands may overcome these dynamic effects and be used to rescue NQO1 function. Thus, we explore here the relation between protein dynamics, stability and functionality of NQO1 polymorphisms using a combination of experimental and computational procedures, including biophysical binding and stability studies, analyses of the local flexibility by proteolysis and mass spectrometry, molecular dynamic simulations and expression studies in cultured cells. The results provided here are critical to understanding NQO1 loss-of-function in cancer patients and for the discovery of new ligands aimed at rescuing NQO1 function by shielding the polymorphic variants from proteasomal degradation.

## Results

### FAD and dicoumarol additively stabilize NQO1 enzymes towards thermal denaturation

**Figure 1:**
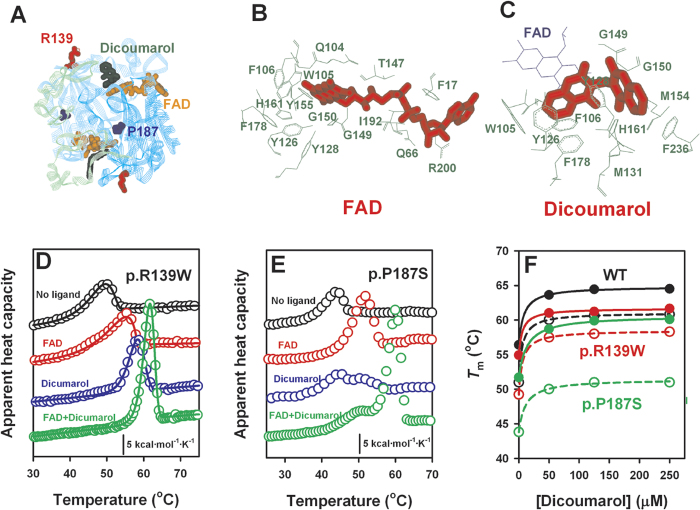
Dicoumarol and FAD mediated stabilization of NQO1 enzymes. (**A**) Structural location of Arg139 (red) and Pro187 (blue) and the ligands FAD (orange) and dicoumarol (dark green) based on the ternary complex determined by X-ray diffraction (PDB: 2F1O^35^). The N-terminal domain (residues 2–217) and C-terminal domain (residues 218–274) are displayed in cyan and light green, respectively; (**B,C**) Binding sites of FAD (B; PDB: 1D4A^36^) and dicoumarol (**C**, PDB: 2F1O^35^), as determined by X-ray diffraction. (**D,E**) DSC profiles of R139W and P187S enzymes in the absence and presence of 100 μM FAD and 250 μM dicoumarol. Lines in panel (**D**) are fits to a two-state kinetic model. (**F**) Dicoumarol concentration dependence of *T*_m_ values for NQO1 enzymes in the absence (open symbols) and presence (closed symbols) of 100 μM FAD. For WT and p.R139W, *T*_m_ values are derived from fits to a two-state kinetic model, while for p.P187S correspond to the maximum of the heat capacity values for the high-temperature transition.

Arg139 and Pro187 are located in solvent exposed loops within the N-terminal catalytic domain (Fig. 1A). These two residues are not directly involved in the binding sites of FAD and/or NADH/dicoumarol (Fig. 1A–C), even though p.P187S strongly decreases NQO1 activity (*k*_cat_ of 0.5–1 s^−1^ for p.P187S *vs*. 100–150 s^−1^ for WT, in three different protein batches) and affinity for FAD^13^,^17^,^20^,^21^ (see Table 1). Nevertheless, addition of FAD causes a strong kinetic and thermal stabilization of all NQO1 enzymes^18^,^20^ (Fig. 1D–F). The p.P187S polymorphism also decreases the binding affinity for dicoumarol by 100-fold compared to WT and p.R139W (Table 1 and Fig. S1). As also seen with FAD, dicoumarol is able to enhance the thermal and kinetic stability of all NQO1 enzymes, and its stabilizing effect is additive to that of FAD (Fig. 1D–F). Therefore, two ligands interacting with different structural regions of NQO1 (FAD primarily with the N-terminal domain, while dicoumarol interacts with residues at both N-terminal and C-terminal domains, Fig. 1B,C) may enhance the global stability of NQO1 polymorphisms *in vitro*.

### Proteolysis supports local changes in NQO1 protein dynamics by cancer-associated polymorphisms and their modulation upon ligand binding

To investigate the potential effects of NQO1 polymorphisms on protein dynamics, possibly leading to reduced conformational stability and impaired catalytic function, we have performed proteolysis studies with thermolysin. First, we have determined the sensitivity of NQO1 variants towards degradation using different concentrations of protease and the effect of added FAD (Fig. S2), since as purified, WT and p.R139W contain significant amounts of FAD bound, while P187S is essentially an apo-protein^18^,^20^. As purified, WT and p.R139W show similar resistance towards proteolysis by thermolysin, while p.P187S is about 100-fold more sensitive. Addition of FAD has little or no effect in the sensitivity of native NQO1 enzymes towards degradation, even though some changes in the proteolysis patterns are observed (Fig. S2). These results agree well with those of a recent study performed with WT and p.P187S using trypsin^21^. Addition of dicoumarol has little effect on the extreme sensitivity of p.P187S towards degradation, while simultaneous addition of FAD and dicoumarol strongly protects p.P187S (Fig. S2).

**Figure 2:**
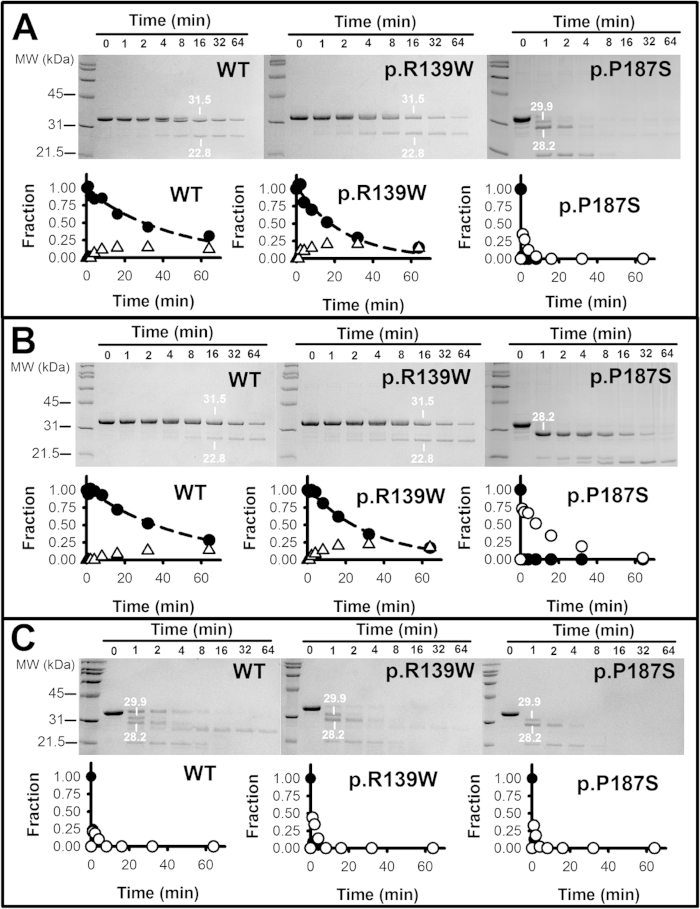
Proteolysis of NQO1 enzymes by thermolysin. Different panels display different experimental conditions: panel (**A**) show data for NQO1 enzymes as purified; panel (**B**) shows NQO1 enzymes in the presence of 100 μM FAD; panel (**C**), apo-NQO1 enzymes. In each panel, SDS-PAGE gels are shown at the upper part, while the time-dependence of native bands (closed circles) and selected intermediates (open triangles for I_22.8_ and open circles for I_28.2_) are shown. Samples identified by HPLC/ESI-MS are indicated in the gels together with their molecular weight determined by this technique.

**Figure 3:**
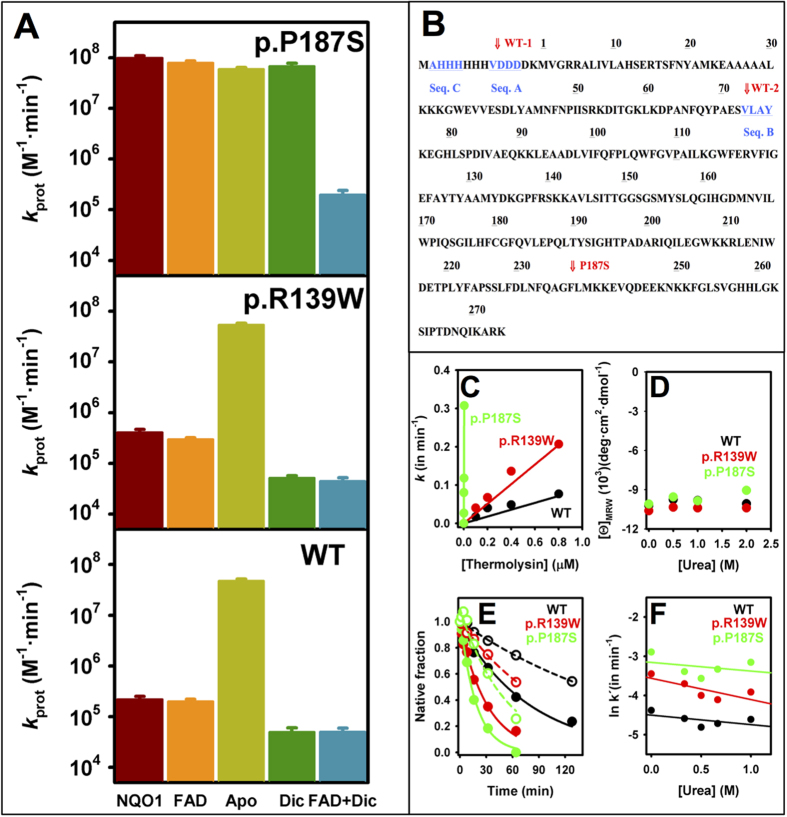
Proteolysis probes the local dynamics at N-terminal and C-terminal domains of NQO1 and the effects of polymorphisms and ligand binding. (**A**) Proteolysis rate constants of different NQO1 enzymes under different experimental conditions: NQO1 (as purified), FAD (in FAD excess), Apo (FAD withdrawn), Dic (in dicoumarol excess) and FAD + Dic (in FAD and dicoumarol excess). (**B**) Cleavage sites mapped onto the sequence of the WT NQO1 construct used in our study. Numbering corresponding to the NQO1 sequence excluding the N-terminal tag. Seqs (**A–C**) are N-terminal sequences determined for selected proteolysis products (highlighted in blue and underlined). The primary cleavage sites are labeled in red. See the main text for additional details. (**C–F**) proteolysis probes the local dynamics of NQO1 proteins. (**C**) The linear response of proteolysis rate constants with protease concentration shows that the proteolysis step is rate-limiting under these conditions; (**D**) Far-UV CD signals at 220 nm after 4 h incubation at selected urea concentrations (protein concentration 6 μM in monomer); (**E**) Proteolysis of NQO1 enzymes as holo-proteins after incubation with 1 M urea for at least two hours (open symbols) or without urea (closed symbols). Thermolysin concentrations were 0.1 μM (WT and p.R139W) and 1 nM (p.P187S). (**F**) Dependence of the proteolysis rate constants at different urea concentrations after correction by the urea effect on thermolysin activity reported by^28^ (*k*´). The equilibrium *m* values are −0.14 ± 0.12 (WT), −0.30 ± 0.15 (p.R139W) and −0.12 ± 0.21 (p.P187S), in kcal·mol^−1^·M^−1^.

To characterize the proteolysis patterns of NQO1 enzymes in the absence and the presence of FAD and dicoumarol, we have performed kinetic analyses by SDS-PAGE in combination with HPLC/ESI-MS and N-terminal sequencing of selected proteolysis products. WT and p.R139W are degraded at similar rates, although degradation of p.R139W is slightly faster, and the proteolysis rates of these two variants are not affected by the addition of FAD (Figs 2A,B and 3A). For these two variants, we observed the accumulation of two partially proteolyzed forms: i) one with a molecular mass of ~31.5 kDa (Table S1; note that our full-length construct is ~32.6 kDa) and a N-terminal sequence (Seq A, site WT-1; Fig. 3B) consistent with the cleavage in the far N-terminal sequence and the release of the his-tag (forming a species with a theoretical mass of 31555 Da). Therefore, this cleavage event essentially leads to the formation of a native full-length NQO1 enzyme; ii) a second form with a size of ~22.8 kDa (from HPLC/ESI-MS analyses, see Table S1) and the N-terminal sequence (Seq B, site WT-2; Fig. 3B), consistent with cleavage between Ser72-Val73 and that would generate a form with a theoretical mass of 22825 Da (in agreement with its experimentally determined mass; Table S1). Therefore, the main proteolysis product of WT and p.R139W by thermolysin is formed upon cleavage at the N-terminal region of NQO1 (named I_22.8_) and contains residues 73–274. This cleavage site differs from those for trypsin and chymotrypsin previously reported in WT NQO1 (occurring at residues 230–240^26^), probably due to different protease specificities.

Proteolysis of p.P187S by thermolysin is very fast and shows a different pattern, with the accumulation of two partially proteolyzed forms (Fig. 2A): a transiently populated form with a mass of 29.9 kDa that leads to the formation of a 28.2 kDa product that is strongly stabilized by FAD (Fig. 2B). This proteolysis pattern also resembles that of p.P187S using trypsin^21^. The 28.2 kDa product has an N-terminal sequence (Seq C in Fig. 3B) consistent with cleavage between residues 1 and 2 of our NQO1 construct, and thus, thermolysin is also cleaving at its C-terminal region. Since thermolysin cleaves at the N-terminus of hydrophobic residues^27^, the cleavage site is either between Gly235-Phe236 (theoretical mass, 28087 Da) or Phe236-Leu237 (theoretical mass, 28234 Da) which we refer to as the P187S site (Fig. 3B) and the intermediate formed as I_28.2_. Therefore, FAD binding seems to strongly affect NQO1 dynamics, but in the case of p.P187S, the far C-terminal region is still highly dynamic and sensitive to proteolysis (in agreement with^21^). Dicoumarol binding strongly protects (by ~400-fold; Fig. 3A) p.P187S towards degradation in the presence of FAD (Figs S2 and S3), supporting the hypothesis that its binding overcomes the dynamical effects of this polymorphism when FAD is also bound. In contrast, dicoumarol binding modestly protects WT and p.R139W (~5-fold), and this stabilization does not depend on the addition of a FAD excess (Fig. 3A). We also noted that, a dicoumarol specific effect on the C-terminal dynamics of p.P187S could contribute to the low affinity of this variant for dicoumarol (Table 1).

Since p.P187S contains low levels of FAD as purified due to its low binding affinity^20^,^21^ (and Table 1), we evaluated the proteolysis kinetic patterns of the three apo-proteins (Fig. 2C). Interestingly, all three NQO1 enzymes show a similar behaviour, being extremely sensitive towards cleavage, and thus they appear to be very dynamic ensembles (Figs 2C and 3A). In the case of WT and p.R139W, removal of FAD leads to a 200-fold increase in the sensitivity towards thermolysin (Fig. 3A), which qualitatively agrees with previous reports on WT NQO1 using trypsin^21^,^26^. Analyses of the proteolysis reactions of apo-NQO1 enzymes at short times revealed a very heterogeneous mixture of proteolysis products (Fig. 2C), but we also detected high levels of the two proteolysis products corresponding to those found in p.P187S, with masses of 29.9 and 28.2 kDa (Table S1). According to these data, and previous proteolysis experiments on apo WT using trypsin^21^,^26^, proteolysis of apo-enzymes support a highly flexible C-terminal domain.

The cleavage site between Ser72-Val73 (site WT-2) is located in a helix and displays average B-factors and low solvent accessibility based on the crystal structures of either WT or p.P187S NQO1 (Fig. S4). In principle, this cleavage site would not be accessible in the native state, and thus, it would require a local unfolding event to become susceptible to proteolysis. In all structures analyzed, the cleavage site for p.P187S (Gly-235-Leu237; site P187S) is found at a more solvent-exposed region with high B-factors (Fig. S4), suggesting that this region would be highly dynamic in the native state. However, caution is required when using the X-ray crystal structure to derive dynamic information on these cleavage sites, and instead, we used dynamic information obtained from molecular dynamic simulations (see next section).

To determine whether cleavage at sites WT-2 and P187S occur through an unfolding event or due to a local fluctuation, we have measured the kinetics of proteolysis in the presence of low urea concentrations^28^. First, we confirmed that, under the experimental conditions, the proteolysis step is rate-limiting for all NQO1 enzymes (Fig. 3C). Then, we selected low urea concentrations (0–1 M) that do not cause significant unfolding based on Far-UV CD measurements (Fig. 3D). These conditions are thus reflecting proteolysis of NQO1 enzymes under near-native conditions. In this scenario, if significant unfolding is required to reach the cleavable state, the proteolysis rate constant will strongly depend on urea concentration (i.e. urea will speed up proteolysis), and the urea concentration dependence of the proteolysis rate constant will yield the equilibrium *m* value between the native and cleavable states, which is expected to be 3.1 kcal·mol^−1^·M^−1^ for the complete unfolding of the NQO1 monomer^29^. Conversely, if a local fluctuation leads to the cleavable state, then the proteolysis rate constants will be essentially independent of urea concentration^28^. In our case, we found that proteolysis was somewhat slowed down by the presence of urea (Fig. 3E), due to the modest inhibition of thermolysin by urea^28^. When corrected for this effect (*k*´ in Fig. 3F), equilibrium *m* values are almost zero (Fig. 3F), supporting the local fluctuation mechanism for the proteolysis of all three holo-NQO1 enzymes.

### Effect of polymorphisms and ligand binding on NQO1 global stability from MD simulations

We have then studied whether the changes in thermal/kinetic stability of NQO1 due to polymorphisms and ligand binding correlate with changes in the average dynamics of NQO1 using molecular dynamics (MD) simulations. In these simulations, the polymorphisms and/or the ligands have no significant effects on the overall secondary structure content (α-helix and β-sheet; Table S2) in agreement with previous experimental spectroscopic analyses^20^. Furthermore, we do not observe major differences in sampled conformations as the maximum RMSD between all systems is below 2 Å.

**Figure 4:**
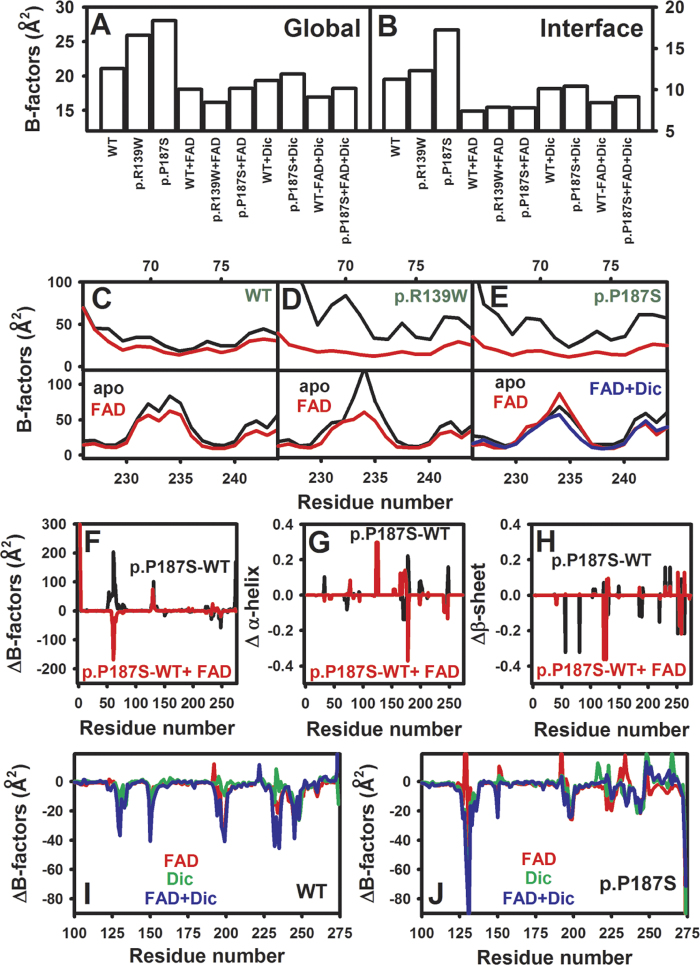
MD simulations support the role of global and local dynamics on the effects of polymorphisms on stability, catalytic function and ligand binding. (**A,B**) Effects on the global (**A**) and dimer interface (**B**) dynamics (B-factors); (**C–E**) Effects of NQO1 polymorphisms, FAD and FAD + dicoumarol binding on the dynamics at the residue level at the vicinity of the N-terminal and C-terminal primary cleavage sites of the NQO1 enzyme. (**F–H**) Difference in dynamics (**F**), α-helix (**G**) and β-sheet (**H**) propensities between p.P187S and WT NQO1 without bound ligands and with FAD bound at the residue level. (**I,J**) Effect of ligand binding (FAD, dicoumarol and FAD + Dicoumarol) on the local dynamics of WT (**I**) and p.P187S (**J**) at the residue level.

Three parameters that refer to the conformational dynamics were analyzed: B-factors (Fig. 4A), dihedral entropies and total vibrational entropies from normal mode analyses (Fig. S5). Average B-factors for the apo-proteins showed a good correlation with their effect on thermal/kinetic stability, with larger average values as the stability decreases. These effects were also observed for dihedral and vibrational entropies, but to a lower extent. Addition of FAD causes to all three proteins a remarkable decrease in the average B-factors (Fig. 4A), consistent with the strong stabilization exerted by FAD in all three variants^20^ (and Fig. 1). Again, the same trend is observed for the dihedral and vibrational (Fig. S5) entropies, but the results are less pronounced. Addition of dicoumarol also reduces the flexibility of apo- and holo-forms of WT and p.P187S (Figs 4A and S5), consistent with its global stabilizing effect on both variants.

Since the NQO1 dimer dissociates prior to the rate-limiting step of thermal denaturation^20^, the stability and flexibility of the monomer-monomer interface must strongly affect the kinetic stability of NQO1. Indeed, the destabilizing effect of polymorphisms and stabilizing effect of FAD binding are associated with large changes in the flexibility of the monomer-monomer interface (Fig. 4B). These changes in flexibility may affect the optimal interactions at the dimer interface, thus contributing to the kinetic stability of NQO1 enzymes.

### Correlation between changes in local dynamics from proteolysis and MD simulations

As native state local fluctuations govern NQO1 proteolysis kinetics, we have used our MD simulations to correlate proteolysis experiments by thermolysin (and trypsin^21^) with changes in flexibility at the residue level. In the case of NQO1, proteolysis kinetics at a certain site depends on the local dynamics of the site as well as on the intrinsic preference of the protease to cleave at that sequence. For these analyses, we have considered a window of about 10 residues around the cleavage site, since the conformation (and dynamics) of 10–12 residues around the cleavage site determines efficient protease binding and cleavage^30^. The flexibility of all three variants around the Ser72-Val73 cleavage site decreases upon FAD binding, whereas only WT and p.R139W also showed a similar decrease in the Gly235-Leu237 cleavage site (Fig. 4C–E). These results are consistent with fast cleavage of holo-p.P187S at the P187S site (Fig. 3A,B; please see^21^ for similar results using trypsin). Similar analyses performed using dihedral entropies showed that FAD binding causes a modest decrease in flexibility at the Ser72-Val73, but no clear effect at the Gly235-Leu237 site (Fig. S5). Simultaneous binding of FAD and dicoumarol caused a decrease in flexibility at the Gly235-Leu237 cleavage site of p.P187S (Fig. 4E), supporting the conclusion that this variant is protected towards proteolysis in the presence of both ligands.

### Changes in local dynamics at the FAD and NADH/Dicoumarol binding sites explain the catalytic impairment of p.P187S

p.P187S binds FAD and dicoumarol (a competitive inhibitor of NADH) with ~10–100 fold lower affinity than the WT enzyme^20^,^21^ (Table 1). To explain these alterations in binding affinity at the structural and dynamic levels, we have compared the flexibility (B-factors) and secondary structure propensities of p.P187S and WT NQO1 from our MD trajectories (Fig. 4F–J). It is worth noting that changes in either the ligand-free or the ligand-bound conformational ensembles are expected to affect the ligand binding affinity.

The p.P187S polymorphism seems to affect the flexibility of different regions important for FAD binding (Figs. 1B and 4F). As apo-protein, this polymorphism increases the flexibility of the region 58–67, which is compensated by FAD binding in p.P187S, and thus causes an energy (entropic) penalty to fix the right interaction of Gln66, Tyr67 and Pro68 with the FAD molecule. A significant increase in flexibility is also found for p.P187S, either as apo- and holo-enzyme, in the region 127–134 including Tyr126 and Tyr128 which are part of the FAD binding site, also possibly affecting the proper interaction with FAD. In the FAD-bound state of p.P187S, we also observed local changes in secondary structure propensities at several regions involved in the FAD binding site (residues 123–127, 170–179 and 241–248; Fig. 4G,H) which may also destabilize the FAD-NQO1 complex in this variant by distorting the FAD binding site.

Binding of dicoumarol has also significant effects on the local dynamics of NQO1 WT and p.P187S (Fig. 4I,J) altering the dynamics of three regions forming the dicoumarol binding site (Fig. 1C), and notably, these changes differ between WT and p.P187S (Fig. 4I,J). Residues 127–136, 149–155 and 230–238 involve regions of high flexibility in the apo-state of WT, which show much lower flexibility upon ligand binding, especially in the ternary (FAD + dicoumarol) complex (Fig. 4I). It is also important to note that in our MD simulations, dicoumarol does not adopt a well-defined binding pose in the absence of FAD, but binding becomes much stronger when FAD is also present. The most noticeable change in p.P187S is found in the region 127–136 (Fig. 4J), which is much more flexible in the apo-state than that for WT. Therefore, this region becomes much more *conformationally restrained* in the ternary complex, and thus, entropically penalizes dicoumarol binding to p.P187S explaining its lower affinity for this inhibitor.

### Rescue of p.P187S in cultured cells requires stabilization of its C-terminal domain

The dynamic alterations caused by p.P187S might be important to understand the low activity and stability of this polymorphism *in vivo*. Since small ligands such as FAD and dicoumarol correct these dynamic alterations *in vitro*, they could be used pharmacologically to rescue p.P187S. We have thus evaluated the effect of riboflavin and dicoumarol in NQO1 protein levels using cell lines endogenously expressing different NQO1 variants: HeLa (homozygotes for WT), HCT 116 (heterozygotes for p.R139W) and Caco-2 cells (homozygotes for p.P187S).

**Figure 5:**
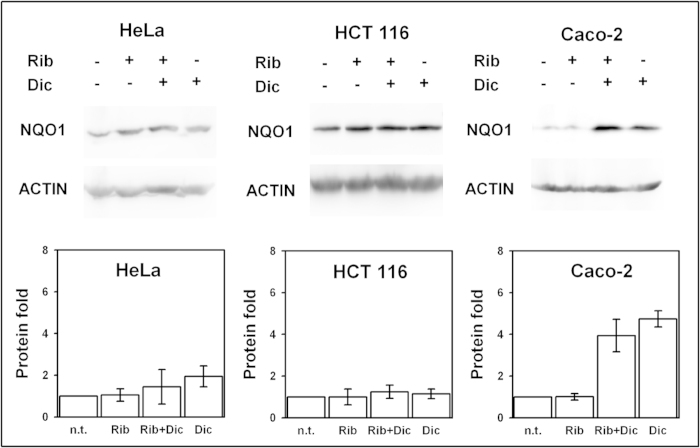
Pharmacological rescue of p.P187S. Effect of riboflavin (Rb) and/or dicoumarol (Dic) on the steady-state NQO1 protein levels in HeLa cells (expressing WT NQO1), HCT 116 cells (heterozygous for p.R139W) and Caco-2 cells (homozygous for p.P187S). Representative western-blots for NQO1 and β-actin in soluble extracts upon treatment with or without riboflavin and/or dicoumarol. The plots show the protein NQO1 levels corrected for β-actin levels and normalized using those from untreated cells. Data are mean ± S.E.M. from three independent experiments.

The steady-state levels of p.P187S in Caco-2 cells are enhanced 4-5 fold in the presence of dicoumarol, while riboflavin alone (a precursor of FAD) has a much lower effect (Fig. 5). Remarkably, these two ligands have small effects on the protein levels of WT (HeLa cells) and p.R139W (HCT 116 cells) (Fig. 5). Since the stability of p.P187S towards proteasomal degradation correlate well with its protein levels in cells, without significant effects on mRNA levels^19^, these results suggest that dicoumarol binding is protecting p.P187S towards proteasomal degradation. Therefore, our results may imply that the intracellular stability of p.P187S strongly depends on the dynamics of its C-terminal domain, which could be therapeutically targeted by ligands binding to its C-terminal domain (such as dicoumarol). Moreover, since intracellular levels of NQO1 are determined to a large extent by the rate of degradation by the 26 S and/or 20 S proteasome^18^,^19^, our results also suggest that the proteasome degrades p.P187S preferentially through its flexible C-terminal end.

## Discussion

The effects of pathogenic genetic variations on protein folding, stability and function are often interpreted using high-resolution crystal structures. However, alterations in protein dynamics are not straightforwardly inferred from this type of structural approach. Particularly, the effect of the p.P187S polymorphism on the crystal structure of NQO1 have been shown to be marginal^21^, and therefore, little insight into the pathogenic mechanisms leading to increased risk of cancer can be extracted from these *static* analyses. By contrast, the present study shows that the pathogenic effects of p.P187S can be studied in-depth using a combination of experimental and computational approaches aimed at correlating changes in protein dynamics, stability and functionality *in vitro* and inside cells. Indeed, the low activity and stability of p.P187S appears to have a dynamic origin, caused by a significant increase in local flexibility at the active site, particularly at the FAD and NADH/substrate binding sites and at the dimer interface. While riboflavin/FAD supplementation may rescue the activity of p.P187S by shifting the equilibrium towards the functional holo-state, the intracellular stability of this polymorphism seems to require a ligand (such as dicoumarol) that overcomes the dynamic alterations at the C-terminal domain, at least in our cell model systems. In the case of p.R139W, dynamic alterations are much weaker thus leading to only modest changes in activity and stability^11^,^20^,^22^, and therefore, the main molecular pathogenic mechanism for this polymorphism is likely to be the skipping of exon 4 (residues 102–139 of the full-length protein) that destroys the binding sites of FAD and the substrate (Fig. 1B,C) rendering an inactive and unstable protein^11^.

A recent study has shown that apo-NQO1 proteins are highly susceptible to proteolysis by trypsin, and in the case of p.P187S, this high susceptibility is not corrected upon FAD binding^21^. These results, in combination with those from NMR spectroscopy, have supported that p.P187S in the apo-state is highly dynamic, while in the holo-state its C-terminal domain remains flexible, which may compromise its catalytic efficiency^21^. Importantly, our present work allows to map changes in protein dynamics at the residue level due to p.P187S and ligand binding, and also to link them with alterations in protein stability (specially at the monomer:monomer interface and the C-terminal domain) and activity. Indeed, p.P187S affects the dynamics of both the C-terminal (as previously shown by^21^) and N-terminal domains, particularly at the FAD and NADH/dicoumarol binding sites, and these dynamic alterations in both domains contribute to its poor catalytic performance and binding affinity for these ligands. We also observe that FAD binding is not sufficient to correct the dynamic alterations at the C-terminal domain *in vitro* (as also shown by^21^) and in eukaryotic cells, and that dicoumarol binding is required for its correction. Our experiments thus support that the dynamics of the C-terminal domain, and its modulations by ligands, determine to a large extent the intracellular stability of p.P187S.

Our results have important implications for the search of pharmacological ligands correcting NQO1 intracellular stability and function, as new therapeutic approaches for those cancer patients bearing the p.P187S polymorphism, similar to those recently proposed to rescue unstable cancer-associated mutants of p53^31^,^32^. Protein degradation by the 20 S proteasome may proceed by either the N- and C-terminus, as well as from internal sequences, and depends on the protein substrate and the local stability of the initiation site recognized by the proteasome^25^. Similarly, degradation by the 26 S proteasome requires the recognition of a polyubiquitin tag and a disordered initiation site, often in the form of a dynamic tail of about 30 amino acids^33^. Therefore, local (rather than global) unfolding rates and/or thermodynamic stabilities may determine the directional preference of proteasomal to degrade proteins (as recently proposed by^34^). Our MD simulations show that the C-terminal end of NQO1 is more flexible than the N-terminal (Fig. S6), which may imply that degradation of the WT protein proceeds preferentially through its C-terminal. Accordingly, the enhanced proteasomal degradation of p.P187S would be linked to its dramatic effect on the C-terminal dynamics. Therefore, in the search for novel pharmacological chaperones to rescue p.P187S, we should take into account their effects on the stability and dynamics of the C-terminal domain.

In conclusion, we provide a comprehensive picture of the structural and dynamic consequences of NQO1 polymorphisms, linking changes in protein dynamics with impaired NQO1 function and FAD binding, particularly for p.P187S, and leading to increased cancer susceptibility (and to lower responses to some chemotherapeutic strategies). Moreover, alterations in protein dynamics can be modulated by small ligands, therefore setting a molecular framework to search for new pharmacological ligands to treat patients bearing these polymorphisms. Additionally, our approach could be used to unravel the pathogenic mechanisms underlying other loss-of-function genetic diseases associated to increased protein turnover, and notably, to dissect the complex effects of mutations and polymorphisms on the conformational and dynamic features of native proteins ultimately causing catalytic and stability defects.

## Materials and Methods

### Protein expression and purification

NQO1 enzymes were expressed in and purified from *Escherichia coli* as N-terminal hexahis-tagged proteins as described^20^. Apo-proteins were obtained upon treatment with potassium bromide as described^20^. NQO1 enzymes were stored in HEPES-KOH 50 mM pH 7.4 at −80 °C and their concentration measured spectrophotometrically as described^20^. Three independent purifications of each NQO1 were used thorough this study, and control experiments were performed to confirm that the results are batch-independent.

### Differential scanning calorimetry (DSC)

DSC studies were performed on a capillary VP-DSC differential scanning calorimeter (Malvern Instruments) with a cell volume of 0.135 mL. Scans were performed at a 1–3 °C·min^−1^ in a temperature range of 5–80 °C using 5–30 μM NQO1 (monomer) in 50 mM HEPES-KOH, pH 7.4. In some experiments, FAD and/or dicoumarol (both from Sigma-Aldrich) were added to a final concentration up to 0.5 mM. Stock solutions of dicoumarol 10 mM were prepared in 0.1 M NaOH.

### Activity measurements

NQO1 activity was measured in 50 mM HEPES-KOH, pH 7.4. A reaction mixture containing recombinant NQO1 and 0.5 mM NADH was incubated at 30 °C for 5 min in a 1 cm path length quartz cuvettes in a thermostatized Agilent 8453 diode-array spectrophotometer. The reaction was triggered by the addition of DCPIP 75 μM as the electron acceptor. Initial reaction rates were determined from changes in A_600nm_ resulting from the reduction of DCPIP and corrected for the non-enzymatic reaction. The NQO1 concentration used varied depending on the variant to ensure linearity over time and protein concentration: 1–2 nM for WT and 25–50 nM for p.P187S. The specific activity was calculated using ε_600_ _nm_ = 21000 M^−1^·cm^−1^ for DCPIP.

### Isothermal titration calorimetry (ITC)

Experiments were performed in a VP ITC_200_ microcalorimeter (Malvern Instruments) with a cell volume of 0.205 mL. Cell samples contained 5 μM NQO1 enzymes (dimer) in HEPES-KOH 50 mM pH 7.4 and were titrated using 0.25 mM dicoumarol by performing 30–50 injections of 0.8–1.2 μL each 180–240 s. Data were corrected for heats of dilution by performing dicoumarol titrations into buffer. After peak integration, binding isotherms were analyzed using a one-type of independent sites model using the software provided by the manufacturer.

### Proteolysis by thermolysin

Thermolysin from *Bacillus thermoproteolyticus rokko* was purchased from Sigma-Aldrich, dissolved in HEPES-KOH 50 mM CaCl_2_ 10 mM pH 7.4, stored at −80 °C and its concentration measured spectrophotometrically using ε_280_ _nm_ = 66086 M^−1^·cm^−1^. Proteolysis was performed at 25 °C in HEPES-KOH 50 mM CaCl_2_ 10 mM pH 7.4 using 20 μM in NQO1 (monomer), and the reaction was initiated by the addition of thermolysin (at a final concentration of 10 pM-1 μM) in a final volume of 200 μL. Proteolysis reactions were performed using the NQO1 proteins as purified or as apo-proteins. In some cases, FAD and/or dicoumarol were added to a final concentration of 100 μM. At different times, 20 μL of the reaction mixture were withdrawn and proteolysis quenched by addition of 5 μL of EDTA 100 mM pH 8, mixed with Laemmli´s buffer and denatured at 95 °C for 5 min. Samples were resolved in 12% acrylamide SDS-PAGE gels, stained with Coomassie Blue and densitometered using ImageJ (http://rsbweb.nih.gov/ij/). After normalization of the intensities using that of the control sample in the absence of thermolysin, the Intensity (*I*) vs. Time (*t*) profiles were analyzed using a single exponential function:


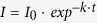


where *k* is the proteolysis rate constant and *I*_0_ the initial intensity.

In some cases, NQO1 samples were incubated with urea (0–1 M) for 4 hours at 25 °C and then proteolysis rates constants were determined as described above. In this case, the observed proteolysis rate constants (*k*) were corrected for the corresponding inhibitory effect of urea on thermolysin at a given urea concentration reported by^28^. These corrected constants (*k*´) were used to determine equilibrium *m* values from the slope of a ln *k*´ vs. [urea] as previously described^28^.

### Limited proteolysis studied by mass spectrometry and N-terminal sequencing

In selected conditions, proteolysis reactions were performed as described above in a volume of 500 μL. After quenching with EDTA, samples were concentrated to 100 μL and submitted to two concentration/dilution cycles with water to a final volume of 100 μL using Vivaspin 10000 Da cut-off filters. These samples were analyzed by high performance liquid chromatography/electrospray-ionization mass spectrometry (HPLC/ESI-MS) at the high resolution mass spectrometry service at the Centro de Instrumentación Científica, University of Granada. HPLC/ESI-MS was performed in a Acquity UPLC system (Waters) using a gradient of water/formic acid (0.1%) and acetonitrile/formic acid (0.1%) using a Acquity UPLC BEH300 C4 column (2.1 × 50 mm; Waters) coupled to a QTOF Synapt62 HDMS (Waters).

For N-terminal sequencing, 100 μL of selected proteolysis mixtures were denatured in Laemmli´s buffer and run in a 12% SDS-PAGE followed by electrotransference to a polyvinylidene difluoride (PVDF) membrane. The membrane was stained with Coomassie blue G-250 and selected bands were cut, destained and equilibrated in water for N-terminal sequencing by the Edman´s method (performed at the service of Protein Chemistry, Centro de Investigaciones Biológicas, Madrid, Spain).

### Circular dichroism spectroscopy

The effect of low urea concentrations (0–2 M) on the conformation of holo-NQO1 enzymes was monitored by circular dichroism (CD) spectroscopy in a Jasco J-710 spectropolarimeter and thermostatized at 25 °C using a Peltier element. Protein samples (6 μM NQO1 monomer) were prepared in HEPES-KOH 50 mM pH 7.4 in the presence of 0–2 M urea, incubated at 25 °C for 4–6 h and their Far-UV CD spectra acquired using 1 mm path length cuvettes. Four scans were measured for each sample in a 210–260 nm range at 100 nm·min^−1^ scan rate and averaged. The corresponding blanks without NQO1 were measured similarly and subtracted.

### Structural analyses based on the NQO1 crystal structures

Structural calculations were performed using the structures 2F1O (for WT NQO1 in the presence of dicoumarol)^35^, 1DXO (for WT NQO1 in the presence of duroquinone)^36^ and 4CF6 (for p.P187S in the presence of Cybacron blue)^21^. Average B-factors were determined for the different protein chains described in the structures and the averages are presented. Solvent accessible surface areas (SASA) of the main-chain and side-chains were calculated using the Shrake-Rupley algorithm^37^ with a radius of 1.4 Å for the solvent probe and the Chothia set for the protein atoms^38^ and a -X-Gly-X- model for the unfolded state using a home-built software kindly provided by Prof. Jose M. Sánchez-Ruíz (Dept. of Physical Chemistry, University of Granada).

### Molecular dynamics simulations

We set up ten independent molecular dynamics simulations of wild-type and mutated NQO1 as apo systems, binary complexes with FAD or dicumarol as well ternary complexes. Systems were prepared in dimeric state on basis of crystal structures of the complex with FAD (PDB: 1D4A^36^) and the ternary complex (PDB: 2F1O^35^). Systems were prepared for simulation using protonate3d and mutated in MOE^39^.

Simulations were performed using the GPU implementation of pmemd in Amber12^40^. Protein residues were parametrized using the Amber forcefield 99SB-ILDN^41^. FAD and dicumarol were parametrized within the Generalized Amber Force Field using additional bonding parameters for FADH^42^. Partial charges for ligands were derived as described earlier^43^. After employing an extensive equilibration protocol^44^, unrestrained systems were sampled for 100 ns in NpT ensemble and 5,000 equal-spaced snapshots were saved to trajectory.

After ensuring thermodynamic and structural stability of simulations, we analyzed resulting conformational ensembles using cpptraj from AmberTools^45^. We analyzed secondary structure elements and B-factors of Cα atoms (derived from root mean squared fluctuations) after a global alignment as a metric for flexibility. Total vibrational entropies were calculated via a normal mode analysis of the covariance matrix of Cα atoms^46^.

Furthermore, we extracted backbone dihedral angles and calculated dihedral entropies based on resulting distributions after employing parameter-free kernel density estimation^47^. Integration over probability densities yields a thermodynamics entropy depicting a metric for local flexibility of the backbone^48^. Dihedral entropies over all three backbone torsions were summed to give a total dihedral entropy per residue. All values are presented as arithmetic average over two dimer sub-units. Interface residues were defined as all residues with an atom closer than 3 Å to the adjacent sub-unit.

### Cell culture and immunoblotting

HeLa, HCT 116 and Caco-2 cell lines were grown in RPMI-1640 with ultraglutamine, supplemented with 10% of FBS (Hyclone) and 1× Antibiotic Antimycotic Solution (Sigma-Aldrich). Cells were incubated with riboflavin 10 μM and/or dicoumarol 100 μM (both from Sigma-Aldrich) for 24 h. Cells were then collected and lysed in RIPA buffer (50 mM Tris-HCl, 150 mM NaCl, 0.1% Triton X-100, 0.1% sodium dodecyl sulphate, 1 mM sodium orthovanadate, 1 mM NaF pH 8) with protease inhibitors (COMPLETE, from Roche) and lysed by freezing-thawing cycles and centrifuged at 18000 g for 15 min. Soluble extracts were collected and the amount of total protein determined by the BCA method (Pierce) using bovine serum albumin as a standard. Approximately 100 μg of total protein were denatured in Laemmli´s buffer at 95 °C for 5 min and loaded into 12% polyacrylamide gels and separated. After transferring to polyvinylidene difluoride membranes (GE Healthcare), immunoblotting was carried out using primary monoclonal antibodies anti-NQO1 and anti-β-actin (Santa Cruz Biotechnology) from mouse at 1:500 and 1:10000 dilutions, respectively. As secondary antibody, we used goat anti-mouse IgG-HRP (Santa Cruz Biotechnology) at 1:1000 dilution. Chemiluminiscent detection of bands was carried out using Clarity™ Western ECL Blotting Substrate (Biorad) and analyzed using a luminescent image analyzer LAS-4000 mini (Fujifilm).

## Additional Information

**How to cite this article**: Medina-Carmona, E. *et al*. Conformational dynamics is key to understanding loss-of-function of NQO1 cancer-associated polymorphisms and its correction by pharmacological ligands. *Sci. Rep*. **6**, 20331; doi: 10.1038/srep20331 (2016).

## Supplementary Material

Supplementary Information

## Figures and Tables

**Table 1:** Binding affinity of NQO1 variants for FAD and dicoumarol determined by ITC.

NQO1 (Ligand)	K_d1_	K_d2_
WT (FAD)	~1 nM	60 nM
p.R139W (FAD)	~3 nM	70 nM
p.P187S (FAD)	400 nM	*N.app*.
WT (Dicoumarol)	122 ± 37 nM	*N.app*.
p.R139W (Dicoumarol)	56 ± 18 nM	*N.app*.
p.P187S (Dicoumarol)	11 ± 3 μM	*N.app*.

Dissociation constants correspond to fittings to a two-sequential binding sites or to a single-set of independent binding sites (*N. app*. indicates a single set of sites model). FAD binding constants are the average of two independent titrations (from^20^) and dicoumarol binding constants are best-fits values ± fitting errors from a single titration (Fig. S1).
