# Erratum: Conformational dynamics is key to understanding loss-of-function of NQO1 cancer-associated polymorphisms and its correction by pharmacological ligands

**DOI:** 10.1038/srep21939

**Published:** 2016-03-04

**Authors:** Encarnación Medina-Carmona, Rogelio J. Palomino-Morales, Julian E. Fuchs, Esperanza Padín-Gonzalez, Noel Mesa-Torres, Eduardo Salido, David J. Timson, Angel L. Pey

Scientific Reports 6: Article number: 2033110.1038/srep20331; published online: 02032016; updated: 03042016

The original version of this Article contained errors in the spelling of the authors Encarnación Medina-Carmona, Esperanza Padín-Gonzalez and Noel Mesa-Torres which were incorrectly given as Medina-Carmona Encarnación, Padín-Gonzalez Esperanza and Mesa-Torres Noel.

In addition, the ‘How to cite this article’ section quoted an incorrect abbreviation for Encarnación Medina-Carmona. These errors have now been corrected in the PDF and HTML versions of the Article.

