# Supplementary material for: Conformational dynamics is key to understanding loss-of-function of NQO1 cancer-associated polymorphisms and its correction by pharmacological ligands
Source: Sci Rep. 2016 Feb 3;6:20331. doi: 10.1038/srep20331 (PMC4738246; doi:10.1038/srep20331)

Conformational dynamics is key to understanding loss-of-function of NQO1 cancer-associated polymorphisms and its correction by pharmacological ligands.

Encarnación Medina-Carmona, Rogelio J. Palomino-Morales, Julian E. Fuchs, Esperanza Padín-Gonzalez, Noel Mesa-Torres, Eduardo Salido, David J. Timson and Angel L. Pey.

Supplementary Information

Table S1. HPLC/ESI-MS analysis of NQO1 proteolysis reactions by thermolysin. The table compiles the main species found in the 40-20 kDa range. Proteolysis were performed in three different conditions: proteins as purified (NQO1), in the presence of a FAD excess (FAD) and as apo-proteins (Apo). The masses corresponding to different species in the proteolysis reactions are shown (in Da). The reaction times were: NQO1 and FAD, WT and p.R139W 16´, p.P187S, 1´; Apo-proteins, 1´; Dicoumarol, WT and p.R139W, 64´, p.P187S, 1´; FAD+dicoumarol, P187S, 16´. Thermolysin concentration was 0.1 μM.

Condition	WT	p.R139W	p.P187S
**NQO1**	31550<br>22825	31580<br>22850	29950<br>28200
**FAD**	31550<br>22825	31585<br>22855	28223
**Apo**	32530<br>29930<br>28210<br>26225<br>21650<br>20845	32540<br>29940<br>28230<br>21500<br>20845	29950<br>28225<br>27673<br>26260<br>20900
**Dicoumarol**	31550<br>26050<br>20500	31580<br>20500	32438<br>29950<br>28200

			26250<br>21200
**FAD+Dicoumarol**	N.Det.	N.Det.	32437<br>31542<br>27335<br>26550

Table S2. Secondary structure content of NQO1 variants in the absence (apo) or presence (holo) of FAD from MD simulations.

	**Apo WT**	**Holo WT**	**Apo p.R139W**	**Holo p.R139W**	**Apo p.P187S**	**Holo p.P187S**
**α-helix**	28.0 %	28.6%	27.6%	28.3%	28.1%	28.9%
**β-sheet**	14.5%	14.3%	14.1%	14.5%	14.2%	14.1%

Figure S1. Isotherms for dicoumarol binding to NQO1 variants by isothermal titration calorimetry. Lines are best-fits to one-type of independent sites binding model. Thermodynamic parameters obtained are compiled in Table 1.

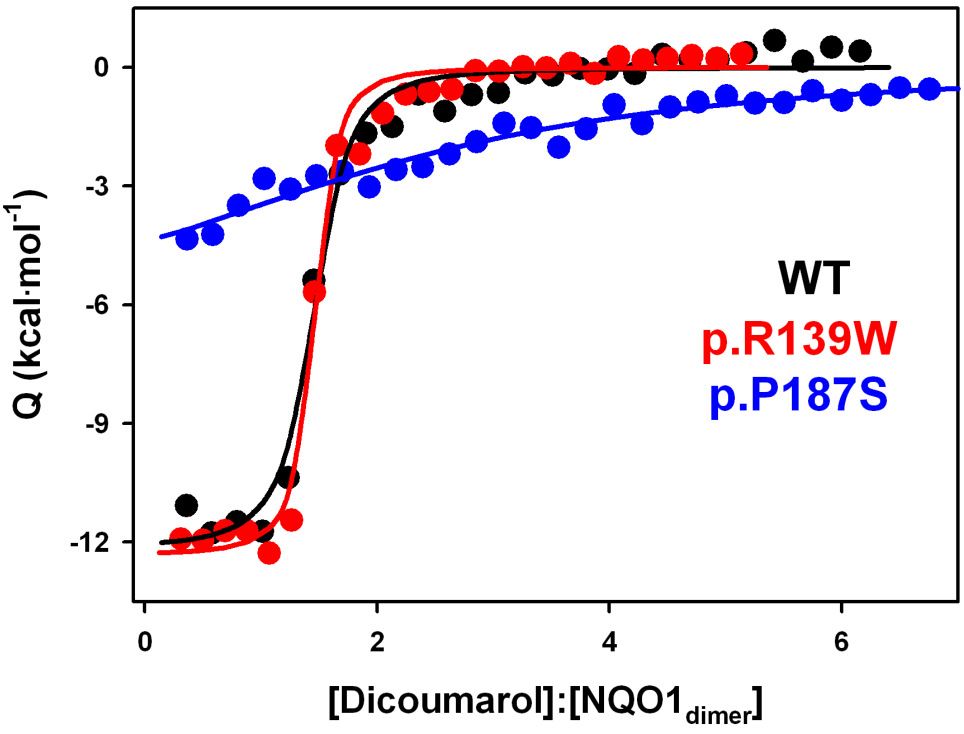

Figure S2. Proteolysis of NQO1 enzymes at different concentrations of thermolysin in the presence of FAD and/or dicoumarol. A) The reactions were performed at 25°C for 10 min, using NQO1 enzymes as purified (-FAD) or incubated with a 5-fold excess of FAD (+FAD; 100 μM). In each panel, representative SDS-PAGE are shown (left) and the corresponding densitometric analyses of the full-length (native) protein (right). B) The reactions are performed for p.P187S as in A, but a 5-fold excess of dicoumarol (100 μM) was also added. SDS-PAGE gels are representative of two independent experiments, and the data shown in the plots are the average from these experiments.

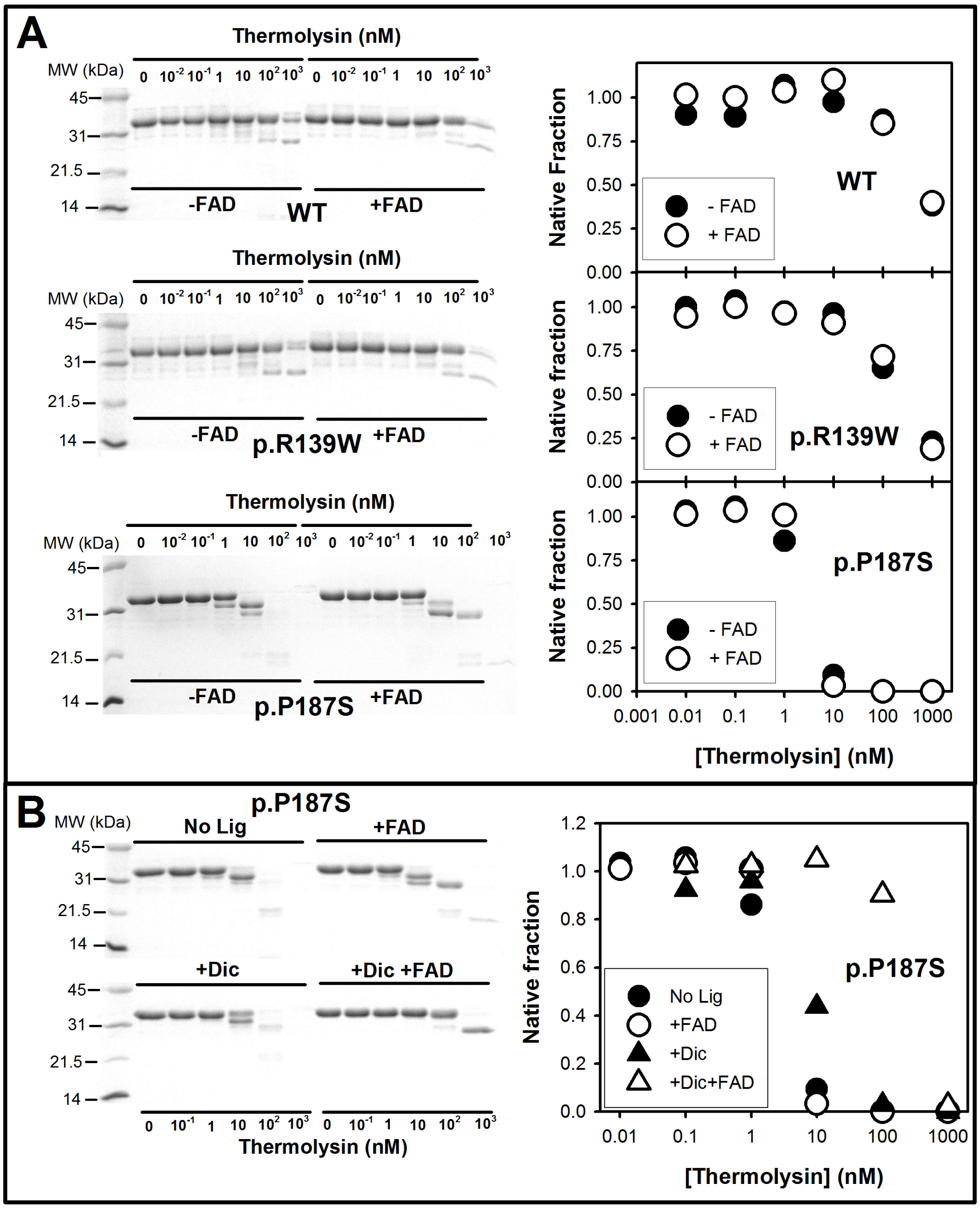

Figure S3. Effect of dicoumarol on the proteolysis pattern of p.P187S. Left) representative kinetic experiments in the absence/presence of dicumarol and FAD. Right) Time dependence population of the native protein (upper plot) or the proteolysis intermediate $I_{28.2}$ (lower plot) in the absence/presence of FAD/Dicoumarol. Experiments performed using 0.1 μM thermolysin, 100 μM ligand and 20 μM p.P187S (in subunit). Data are from two independent experiments.

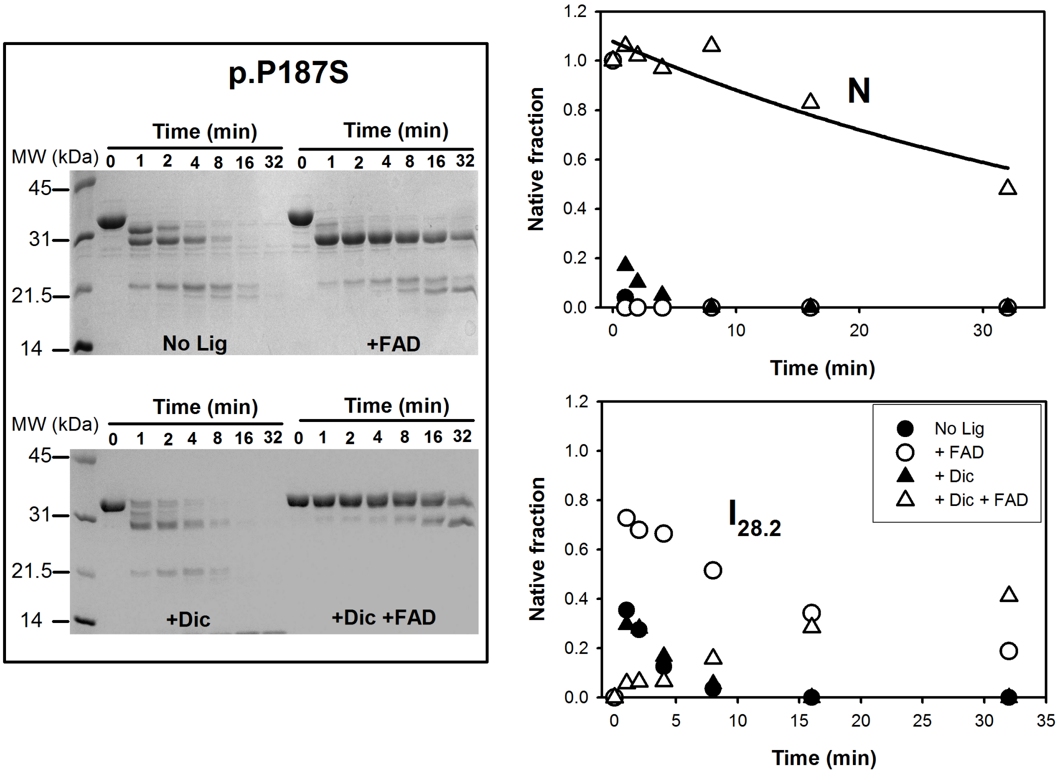

Figure S4. B-factors and solvent accesibilities of cleavage sites (residues 72-73 and 235-237) experimentally determined for WT (in blue, with dicoumarol; in green, with duroquinone) and p.P187S (in red, with Cybacron blue) NQO1. A-C) B-factors (in Å^2) corresponding to the crystal structures of NQO1 WT (PDB: 2F1O and 1DXO) and p.P187S (PDB:4CF6) along the protein sequence (panel A), highlighting those values in the proximity of the cleavage sites (panels B and C). The horizontal solid lines show the average value for the entire sequence ±1 s.d. (dashed lines). D-E) fraction of solvent accesible surface area (SASA) for the main chain and side chains considering a –Gly-X-Gly- tripeptide for the unfolded state (panel D), highlighting those values in the proximity of the cleavage sites (panels E and F).

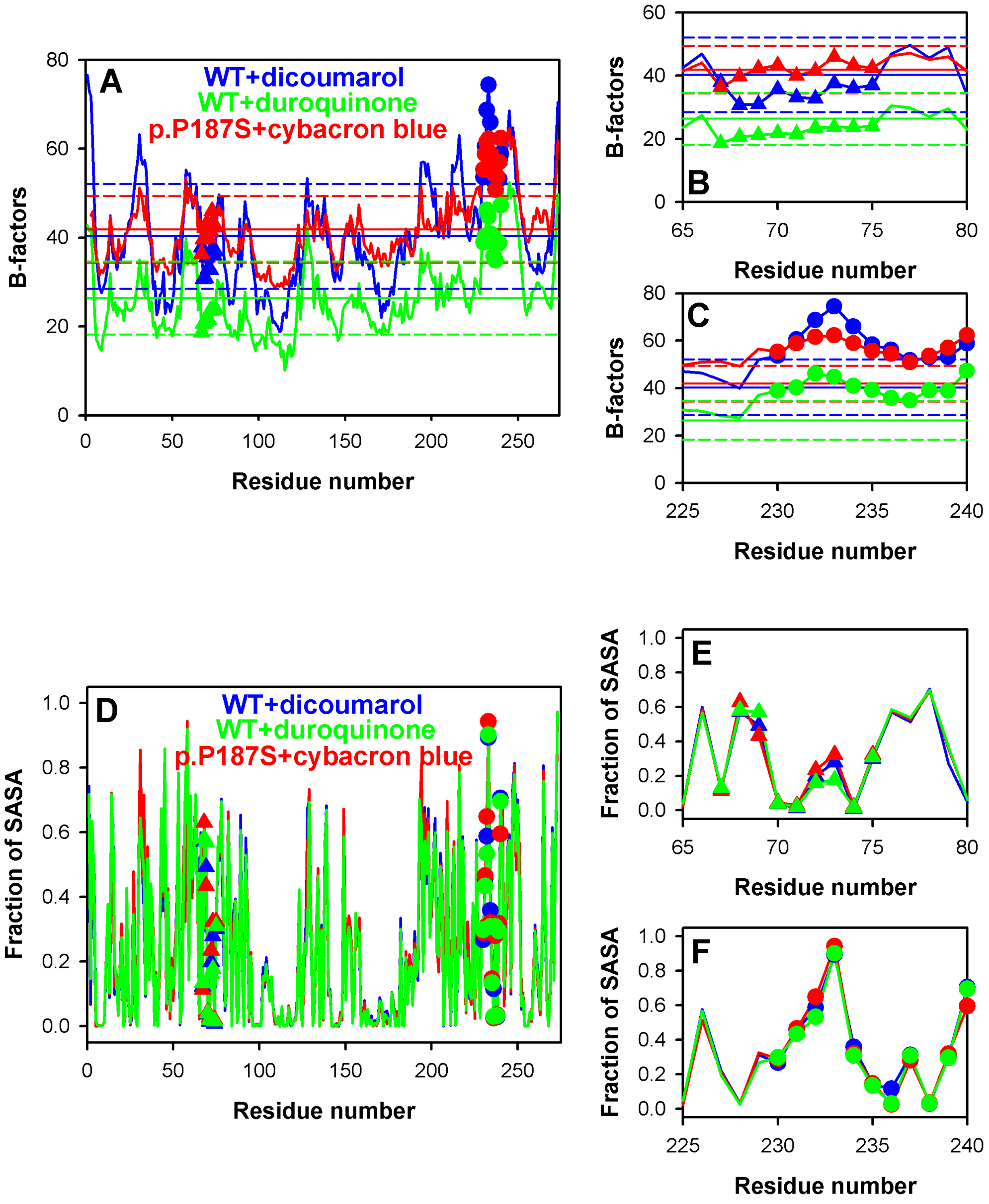

Figure S5. Dihedral and vibrational entropies determined from MD simulations. A-B) average dihedral entropies for the entire protein (A) and the monomer-monomer interface (B); C) vibrational entropies for the entire protein; D-F) dihedral entropies in the vicinity of primary thermolysin cleavage sites (upper panels, Ser72-Val73; lower panel, Gly235-Leu237) as apo-proteins and in the presence of FAD; G) difference in dihedral entropies between WT and p.P187S variants as apo-proteins and in the presence of FAD.

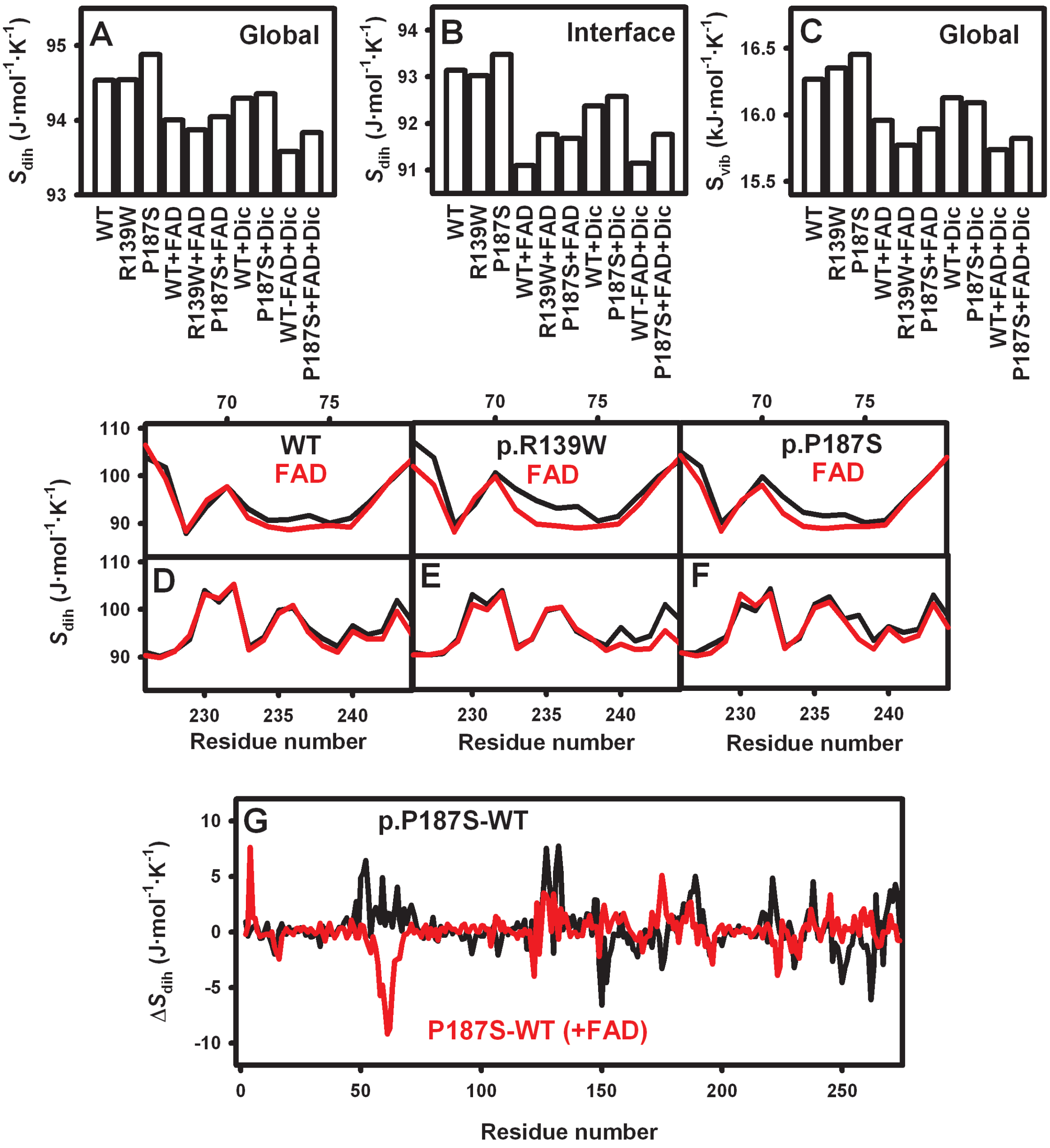

Figure S6. MD simulations support that the C-terminal end of NQO1 variants is more flexible. Data for apo-proteins (black) and holo-proteins (+FAD, red) is shown.

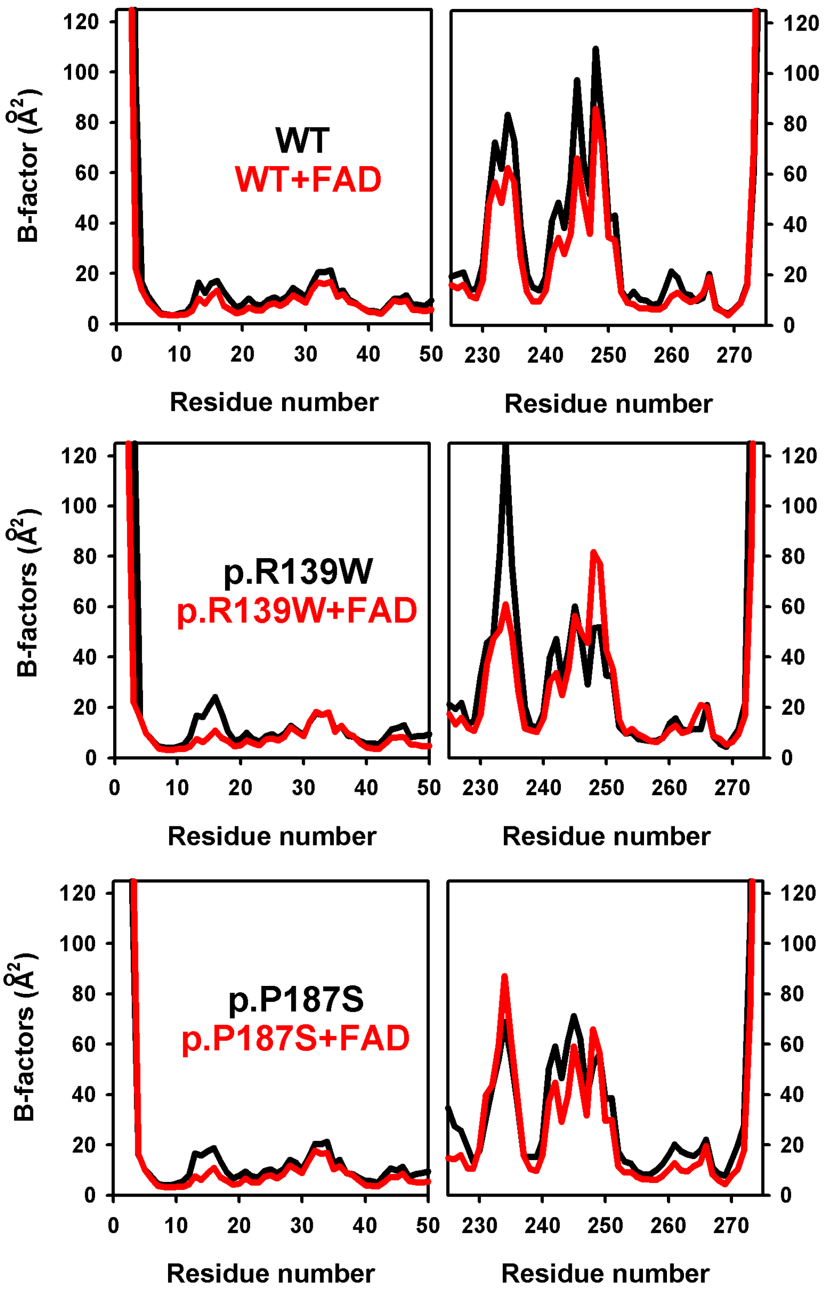

Supplement: Supplementary Information [file srep20331-s1.pdf]
